# Relation of infarction location and volume to vertigo in vertebrobasilar stroke

**DOI:** 10.1002/brb3.1564

**Published:** 2020-02-05

**Authors:** Ahmed Mohamed Elhfnawy, Mervat Abd El‐Raouf, Jens Volkmann, Felix Fluri, Doaa Elsalamawy

**Affiliations:** ^1^ Department of Neurology University Hospital Würzburg Würzburg Germany; ^2^ Department of Neurology University Hospital of Alexandria Alexandria Egypt; ^3^ Department of Neurology University Hospital of Essen Essen Germany

**Keywords:** brain stem, cerebellum, infarction volume, stroke, vertebrobasilar insufficiency, vertigo

## Abstract

**Objective:**

Vertigo is a common presentation of vertebrobasilar stroke. Anecdotal reports have shown that vertigo occurs more often in multiple than in single brainstem or cerebellar infarctions. We examined the relation between the location and volume of infarction and vertigo in patients with vertebrobasilar stroke.

**Methods:**

Consecutive patients with vertebrobasilar stroke were prospectively recruited. The infarction location and volume were assessed in the diffusion‐weighted magnetic resonance imaging.

**Results:**

Fifty‐nine patients were included, 32 (54.2%) with vertigo and 27 (45.8%) without vertigo. The infarction volume did not correlate with National Institute of Health Stroke Scale (NIHSS) score on admission (Spearman *ρ* = .077, *p* = .56) but correlated with modified Rankin Scale (*ρ* = .37, *p* = .004) on discharge. In the vertigo group, the proportion of men was lower (53.1% vs. 77.8%, *p* = .049), fewer patients had focal neurological deficits (65.6% vs. 96.3%, *p* = .004), patients tended to present later (median [IQR] was 7.5 [4–46] vs. 4 [2–12] hours, *p* = .052), numerically fewer patients received intravenous thrombolysis (15.6% vs. 37%, *p* = .06), and the total infarction volume was larger (5.6 vs. 0.42 cm^3^, *p* = .008) than in nonvertigo group. In multivariate logistic regression, infarction location either in the cerebellum or in the dorsal brainstem (odds ratio [OR] 16.97, 95% CI 3.1–92.95, *p* = .001) and a total infarction volume of >0.48 cm^3^ (OR 4.4, 95% CI 1.05–18.58, *p* = .043) were related to vertigo. In another multivariate logistic regression, after adjusting for age, sex, intravenous thrombolysis, serum level of white blood cells, and atrial fibrillation, vertigo independently predicted a total infarction volume of >0.48 cm^3^ (OR 5.75, 95% CI 1.43–23.08, *p* = .01).

**Conclusion:**

Infarction location in the cerebellum and/or dorsal brainstem is an independent predictor of vertigo. Furthermore, larger infarction volume in these structures is associated with vertigo. A considerable proportion of patients with vascular vertigo present without focal neurological deficits posing a diagnostic challenge. National Institute of Health Stroke Scale is not sensitive for vertebrobasilar stroke.

## Graphical abstract

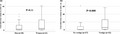

## 1 INTRODUCTION

Dizziness is a major public health problem and an independent predictor of increased mortality (Corrales & Bhattacharyya, 2016). Dizziness and vertigo are more common among women and elderly population (Neuhauser et al., 2008; Rieger et al., 2014). Affected patients do not usually receive the adequate medical attention and are more likely to consult a general practitioner or an internist rather than a neurologist or an ear, nose, and throat (ENT) specialist (Neuhauser et al., 2008). Stroke is the underlying etiology in 17%–25%, and 4% of patients presenting with acute onset isolated vertigo (Norrving, Magnusson, & Holtas, 1995; Zuo et al., 2018) and dizziness (Navi et al., 2012), respectively. Of note, 18.9% of patients with vertebrobasilar stroke suffer from vertigo in comparison with only 1.7% of those with stroke in the anterior circulation (Tao et al., 2012). Lesions affecting the following structures are related to the development of vascular vertigo: vestibular nuclei in the dorsolateral portion of the rostral medulla, nucleus prepositus hypoglossi in the dorsal brainstem, dorsal insular cortex as well as cerebellar tonsil, flocculus, nodulus, and inferior cerebellar peduncles (Kattah, Talkad, Wang, Hsieh, & Newman‐Toker, 2009; Kerber, Brown, Lisabeth, Smith, & Morgenstern, 2006; Kim, Kim, & Kim, 2017; Neuhauser et al., 2008; Rieger et al., 2014; Saber Tehrani et al., 2014). However, one study showed that more than one‐fifth of patients with isolated lateral medullary infarction have no vertigo and patients with lateral medullary infarction plus additional extralateral medullary infarctions are more commonly associated with vertigo than those with isolated lateral medullary infarctions (Kang et al., 2018). Another study reported vertigo in all patients with multiple unilateral pontine infarctions and in less than half of those with a single pontine infarction (Kumral, Bayulkem, & Evyapan, 2002). Moreover, small lesions ≤10 mm in axial diameter were detected in only 14% of patients with acute vascular vertigo (Saber Tehrani et al., 2014). We aimed to examine the relationship of infarction volume and location to vertigo in patients diagnosed with a vertebrobasilar stroke.

## 2 METHODS

### 2.1 Study design

Consecutive patients admitted to the Department of Neurology (University Hospital of Würzburg) with the diagnosis of vertebrobasilar stroke were prospectively recruited between February and October 2018. Patients were included only if they could communicate the presence or absence of vertigo, and a magnetic resonance imaging (MRI) could be done within 4 days of admission and showed brain infarction. National Institute of Health Stroke Scale (NIHSS) on admission and modified Rankin Scale (mRS) and Barthel index on discharge were used as clinical scales. Patients were stratified into two groups: group 1 with vertigo (vertigo +) and group 2 with no vertigo (vertigo −). Vertigo was defined according to the definition of the Bárány Society as follows: “the feeling of self‐motion, when no self‐motion is occurring” (Bisdorff, Von Brevern, Lempert, & Newman‐Toker, 2009).

### 2.2 MRI imaging

Magnetic resonance imaging scanners with a field strength of 3‐Tesla were used according to our standardized stroke acquisition protocols with a slice thickness of 5 mm and an interslice gap of 0.5 mm. The infarction location and volume were assessed in the strong (*b* = 1,000) diffusion‐weighted images (DWI) in our picture archiving and communication system (PACS) by a single investigator (AME), who was nonblinded to the clinical data. The infarction was manually delineated in our PACS to obtain the infarction area in each slice separately. The latter was multiplied by the slice thickness and interslice gap. Finally, the sum of all slices was calculated to obtain the infarction volume (Inaba et al., 2017; Jung, Kwon, Lee, & Kang, 2010).

### 2.3 Statistical analyses

Qualitative data were expressed in absolute values and percentages, while quantitative data were expressed using median and range. To check for normality, we used Q–Q plot, histogram, and the Shapiro–Wilk test. Univariate statistical tests were conducted for categorical data using chi‐squared test, and if *n* < 5, Fisher's exact test was used. For continuous data, we used the Mann–Whitney *U* test. Spearman coefficient was used to analyze correlations. To calculate the cutoff infarction volume value for the occurrence of vertigo, a receiver operating curve (ROC) was used. We chose the points closest possible to the upper left corner to get cutoff values with high sensitivity. An area under the curve (AUC) > 0.5 indicates better prediction, and values closer to 1 indicate more accurate prediction. Univariate binary logistic regression analysis was performed to measure the strength of association, measured as OR (95% CI), between the occurrence of vertigo and other possibly related variables. To adjust for age and sex, we conducted a multivariate logistic regression with inclusion method. In this model, we included variables found in the univariate model with *p* < .1. We tested the fitness of this model using a Hosmer–Lemeshow “goodness‐of‐fit” test. Data were analyzed in SPSS software package version 25 (SPSS). *p*‐values < .05 were considered statistically significant.

## 3 RESULTS

### 3.1 Baseline characteristics

**Figure 1:**
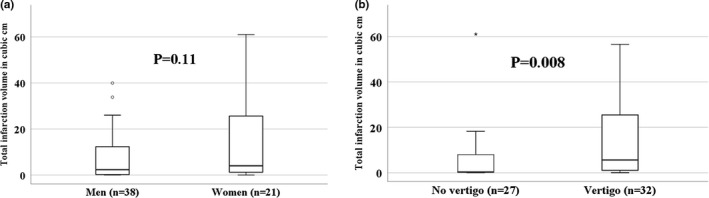
(a) Total infarction volume among men and women (*p* = .11). (b) Total infarction volume among vertigo (+) patients versus vertigo (−) patients (*p* = .008)

Fifty‐nine patients were included in the study. Baseline characteristics are illustrated in Table 1. There was a statistically significant less proportion of men among vertigo (+) patients in comparison with vertigo (−) patients (53.1% vs. 77.8%, respectively, *p* = .049). Otherwise, no statistically significant difference was found between the baseline characteristics of the two groups. The median (IQR) infarction volume among women was 3.99 (0.92–26.86) cm^3^ versus 2.32 (0.21–12.35) cm^3^ among men (*p* = .11, Figure 1a).

**Table 1:** Baseline characteristics

Characteristic	Vertigo (−), *n* = 27	Vertigo (+), *n* = 32	*p*‐value
Age in years, median (IQR)	66 (55–77)	70 (54–79)	.88
Women, *n* (%)	6 (22.2)	15 (46.9)	.049*
Active smoking, *n* (%)	6 (22.2)	6 (18.8)	.74
Hypertension, *n* (%)	22 (81.5)	26 (81.3)	.98
Diabetes, *n* (%)	2 (7.4)	5 (15.6)	.44
Atrial fibrillation, *n* (%)	6 (22.2)	9 (28.1)	.6
History of previous stroke, *n* (%)	7 (25.9)	4 (12.5)	.32
HbA1c%, median (IQR)	5.6 (5.3–6.2)	5.6 (5.3–5.8)	.82
LDL‐Cholesterol (mg/dl), median (IQR)	108 (83–121)	113.5 (84.8–147)	.36
Headache, *n* (%)	6 (24)	14 (45.2)	.1
Absence of focal neurological deficits, *n* (%)	1 (3.7)^c^	11 (34.4)	.004*
NIHSS on admission, median (IQR)	2 (1–5)	2 (0–4)	.16
Good outcome on discharge, *n* (%)^a^	22 (81.5)	25 (78.1)	.75
Onset of symptoms till presentation in hours, median (IQR)^b^	4 (2–12)	7.5 (4–46)	.052
Bilateral infarction, *n* (%)	5 (18.5)	7 (21.9)	.75
Infarction location, *n* (%)			
Cerebellum or brainstem	14 (51.9)	30 (93.8)	<.001*
Cerebellum or dorsal brainstem	11 (40.7)	29 (90.6)	<.001*
Cerebellum	9 (33.3)	26 (81.3)	<.001*
Cerebellar tonsil	2 (7.4)	15 (46.9)	.001*
Cerebellar nodulus	1 (3.7)	7 (21.9)	.06
Dorsal brainstem	3 (11.1)	7 (21.9)	.32
Total infarction volume in cm^3^, median (IQR)	0.42 (0.14–8.4)	5.6 (0.98–25.5)	.008*
Total infarction volume of >0.48 cm^3^, *n* (%)	12 (44.4)	27 (84.4)	.002*
Volume of infarctions located in the cerebellum >0.36 cm^3^, *n*/*N* (%)	2/9 (22.2)	25/26 (96.2)	<.001*
Volume of infarction located in the cerebellum and/or brainstem, median (IQR)	0.24 (0.11–0.38) (*n* = 14)	5.6 (0.77–24.01) (*n* = 30)	<.001*
Volume of infarction located in the cerebellum, median (IQR)	0.26 (0.12–4.92) (*n* = 9)	9.09 (2.4–25.26) (*n* = 26)	.001*
Volume of infarction located in the brainstem, median (IQR)	0.11 (0.05–0.27) (*n* = 7)	0.10 (0.05–0.5) (*n* = 9)	.92
Intravenous thrombolysis, *n* (%)	10 (37)	5 (15.6)	.06

Abbreviations: HbA1c, hemoglobin A1c; IQR, interquartile range; LDL‐cholesterol, low‐density lipoprotein cholesterol; NIHSS, National Institute of Health Stroke Scale.

a Good outcome on discharge was defined as modified Rankin Scale ≤ 2 on discharge.

b In patients with wake‐up stroke or those found with stroke, the time, when the patient was found with stroke, was used to denote the time of symptom onset.

c The patient presented with transient episodes of blurring vision in both eyes. In addition, the patient had nausea and vomiting without vertigo. MRI brain showed a small right cerebellar infarction.

* Statistically significant results.

### 3.2 Relation between infarction location and volume and the presence or absence of vertigo

**Figure 2:**
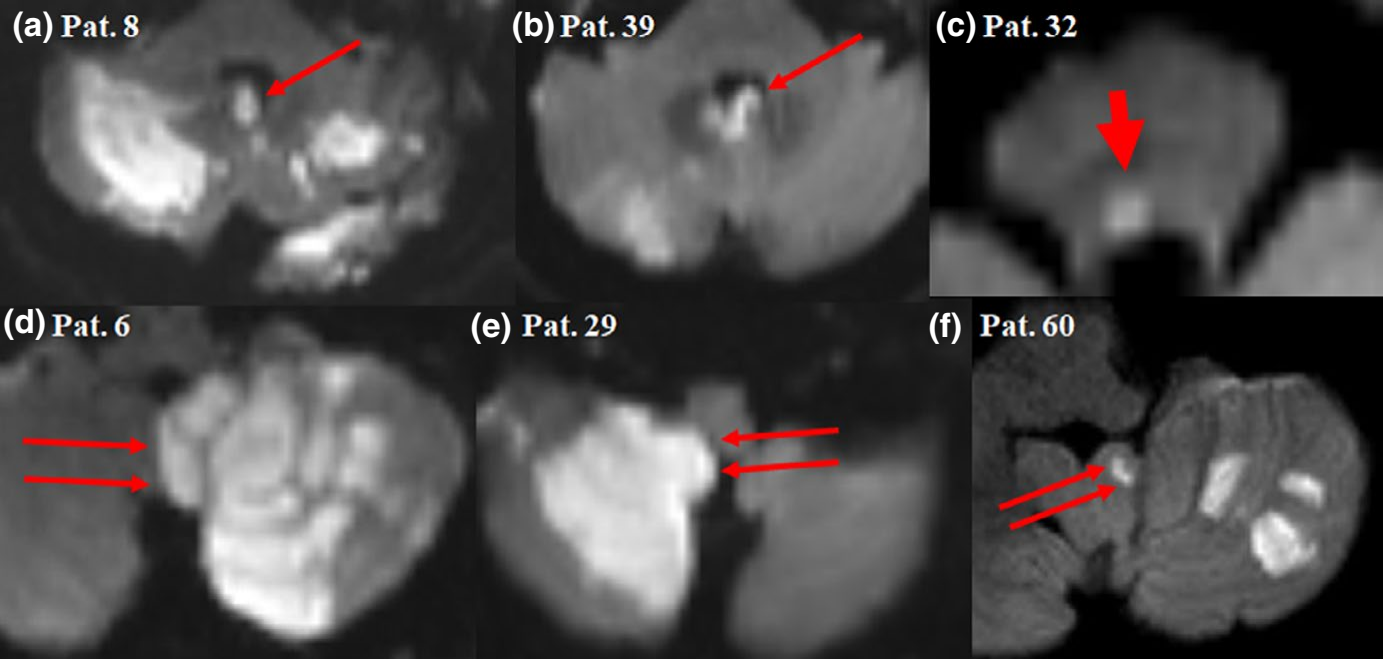
Diffusion‐weighted magnetic resonance imaging showing examples of patients with vertebrobasilar stroke having vertigo. (a) and (b) affection of the nodulus (long thin arrow), (c) affection of the dorsal pons, probably in the nucleus prepositus hypoglossi (short thick arrow), (d) through (f) affection of the cerebellar tonsil (double long thin arrows). Note the large infarction size in comparison with Figure 3

**Figure 3:**
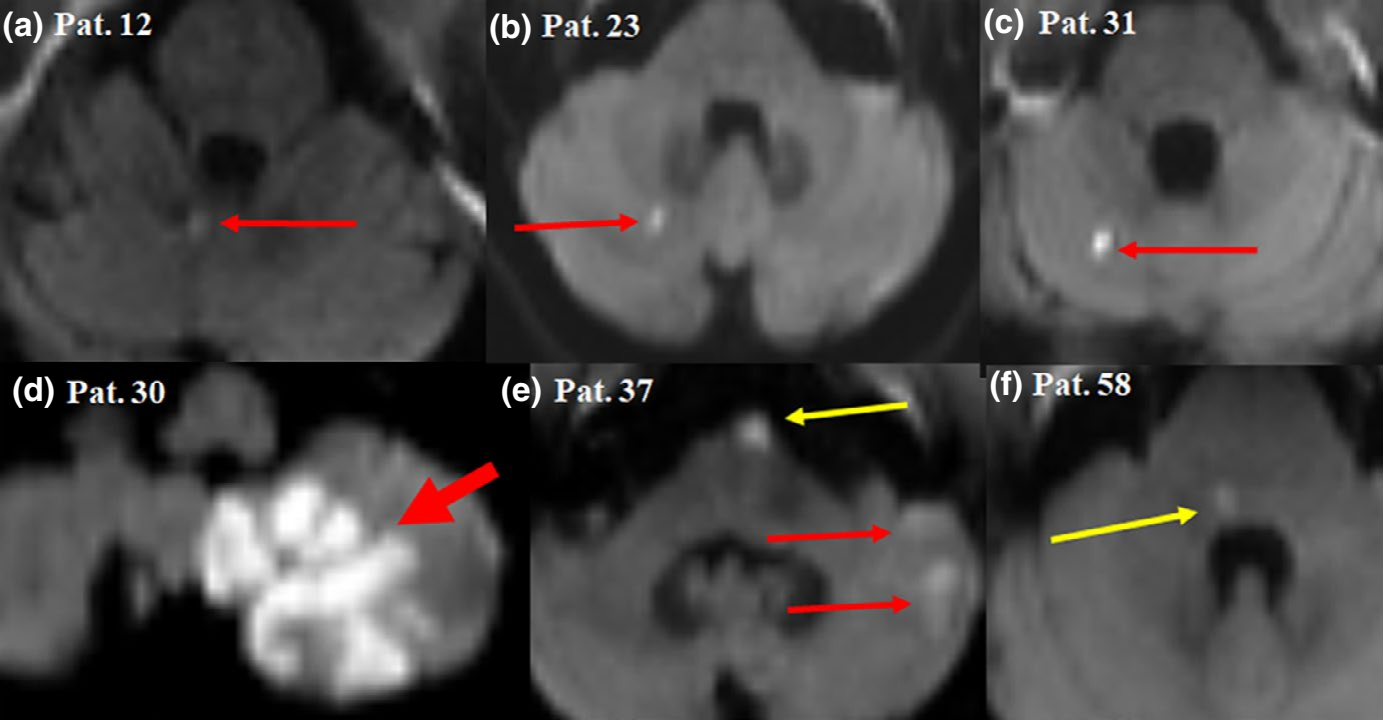
Diffusion‐weighted magnetic resonance imaging showing examples of patients with vertebrobasilar stroke without vertigo. (a) Minute infarction in the vermis (long thin arrow), (b) and (c) minute infarction in the cerebellar hemisphere (long thin arrow), (d) large infarction affecting the cerebellar hemisphere and tonsil (short thick arrow), (e) multiple infarctions affecting the ventral pons (long thin yellow arrow) and the cerebellar hemisphere (long thin red arrow), (f) minute infarction affecting the dorsal pons (long thin yellow arrow). Note the small size of the infarctions in comparison with Figure 2

**Figure 4:**
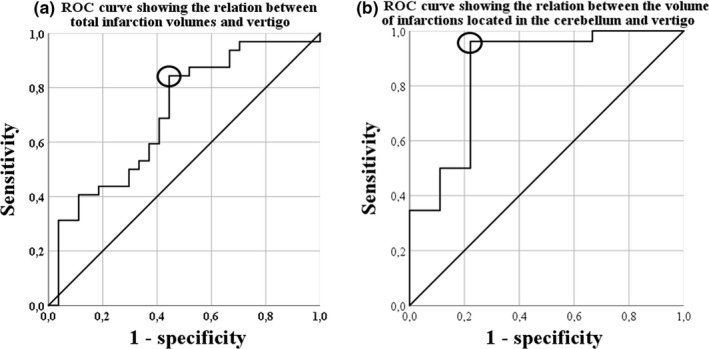
(a). Receiver operating curve (ROC) showing the relation between the total infarction volumes and vertigo: A cutoff volume of >0.48 cm^3^ for all infarctions was associated with vertigo with sensitivity of 84% and specificity of 56% (indicated by a circle); AUC (95% CI) = 0.7 (0.57–0.84), *p* = .008, (b) receiver operating curve (ROC) showing the relation between the volume of infarctions located in the cerebellum and vertigo: A cutoff volume of >0.36 cm^3^ for infarctions located in the cerebellum was associated with vertigo with sensitivity of 96% and specificity of 78% (indicated by a circle); AUC (95% CI) = 0.86 (0.69–1.0), *p* = .002

An infarction location either in the cerebellum or in the dorsal brainstem was significantly more common among vertigo (+) patients in comparison with vertigo (−) patients (90.6% vs. 40.7%, respectively, *p* < .001). The total infarction volume was much larger among vertigo (+) patients with a median of 5.6 cm^3^ versus 0.42 cm^3^ among vertigo (−) patients (*p* = .008, Figure 1b). This difference was even more evident for infarctions located in the cerebellum with a median of 9.09 versus 0.26 cm^3^ for vertigo (+) patients in comparison with vertigo (−) patients, respectively. Figures 2 and 3 show different examples for patients with vertigo (+) and vertigo (−) patients with vertebrobasilar stroke. Using a ROC curve, a cutoff volume of >0.48 cm^3^ for all infarctions was found to be associated with vertigo with sensitivity of 84% and specificity of 56%; AUC (95% CI) = 0.7 (0.57–0.84), *p* = .008 (Figure 4a). Furthermore, a cutoff volume of >0.36 cm^3^ for infarctions located in the cerebellum was found to be associated with vertigo with sensitivity of 96% and specificity of 78%; AUC (95% CI) = 0.86 (0.69–1.0), *p* = .002 (Figure 4b). In an age‐ and sex‐adjusted multivariate binary logistic regression analysis, an infarction location either in the cerebellum or in the dorsal brainstem and an infarction volume of >0.48 cm^3^ were found to have an OR (95% CI) of 16.97 (3.1–92.95), *p* = .001 and 4.4 (1.05–18.58), *p* = .043, respectively to be associated with vertigo as shown in Table 2. The Hosmer–Lemeshow “goodness‐of‐fit” test showed a nonsignificant difference between the observed and expected results with *p* = .88. In another multivariate logistic regression, after adjusting for age, sex, intravenous thrombolysis, serum level of white blood cells, and atrial fibrillation, vertigo independently predicted a total infarction volume >0.48 cm^3^ (OR 5.75, 95% CI 1.43–23.08, *p* = .01) as shown in Table 3 (Hosmer–Lemeshow “goodness‐of‐fit” test showed a *p*‐value of .2).

**Table 2:** Predictors of vertigo in the binary logistic regression models

Characteristic	Univariate regression analysis			Multivariate regression analysis		
	OR	95% CI	*p*	OR	95% CI	*p*
Age in years	0.99	96–1.03	.72	0.96	0.9–1.02	.17
Women	3.09	0.99–9.68	.053	3.8	0.79–18.47	.1
Active smoking	0.81	0.23–2.87	.74			
Hypertension	0.99	0.26–3.67	.98			
Diabetes	2.32	0.41–13.03	.34			
Atrial fibrillation	1.37	0.42–4.5	.61			
Previous stroke	0.41	0.11–1.58	.2			
HbA1c (%)	1	0.55–1.8	1			
LDL‐cholesterol (mg/dl)	1.01	0.99–1.02	.51			
White blood cells*1,000/µl	1.08	0.9–1.29	.41			
CRP (mg/dl)	0.71	0.44–1.16	.17			
ESR in the first hour (mm)	0.99	0.96–1.02	.62			
Total infarction volume of >0.48 cm^3^	6.75	1.99–22.85	.002*	4.4	1.05–18.58	.043*
Infarction affecting the cerebellum or dorsal brainstem	14.06	3.42–57.88	<.001*	16.97	3.1–92.95	.001*
H‐L test^a^						0.88

Abbreviations: CRP, C‐reactive protein; ESR, erythrocyte sedimentation rate; HbA1c, hemoglobin A1c; LDL‐cholesterol, low‐density lipoprotein cholesterol.

a Hosmer–Lemeshow “goodness‐of‐fit” test for the multivariate regression analysis showed a nonsignificant *p*‐value (*p* = .88) for the difference between our observed results and the expected results. The nonsignificant *p*‐value for this test means better fit of the model (the higher the value, the better the fit).

* Statistically significant.

**Table 3:** Factors associated with a total infarction volume >0.48 cm^3^ in the binary logistic regression models

Characteristic	Univariate regression analysis			Multivariate regression analysis		
	OR	95% CI	*p*	OR	95% CI	*p*
Age in years	1	0.96–1.04	.95	0.98	0.93–1.04	.51
Women	3.09	0.87–10.96	.08	1.59	0.31–8.28	.58
Vertigo	6.75	1.99–22.85	.002*	5.75	1.43–23.08	.01*
Active smoking	1.03	0.27–3.96	.96			
Hypertension	1.14	0.29–4.49	.85			
Diabetes	0.65	0.13–3.2	.6			
Atrial fibrillation	4.5	0.9–22.4	.07	4.67	0.77–28.31	.09
Previous stroke	0.88	0.22–3.43	.85			
HbA1c (%)	1.5	0.71–3.16	.28			
LDL‐cholesterol (mg/dl)	1	0.99–1.02	.57			
White blood cells*1,000/µl	1.22	0.97–1.54	.09	1.23	0.93–1.62	.14
CRP (mg/dl)	0.84	0.54–1.3	.44			
ESR in the first hour (mm)	1	0.96–1.03	.91			
Intravenous thrombolysis	0.33	0.1–1.1	.07	0.72	0.16–3.14	.66
H‐L test^a^						.2

Abbreviations: CRP, C‐reactive protein; ESR, erythrocyte sedimentation rate; HbA1c, hemoglobin A1c; LDL‐cholesterol, low‐density lipoprotein cholesterol.

a Hosmer–Lemeshow “goodness‐of‐fit” test for the multivariate regression analysis showed a nonsignificant *p*‐value (*p* = .2) for the difference between our observed results and the expected results. The nonsignificant *p*‐value for this test means better fit of the model (the higher the value, the better the fit).

* Statistically significant.

### 3.3 Vertigo and focal neurological deficits

In the vertigo group, fewer patients had focal neurological deficits (65.6% vs. 96.3%, *p* = .004). Therefore, there was a tendency for vertigo (+) patients to have a delayed presentation in comparison with vertigo (−) patients; median (IQR) was 7.5 (4–46) hours compared to 4 (2–12) hours, respectively (*p* = .052). Moreover, only 5/32 (15.6%) of the patients in the vertigo (+) group received intravenous thrombolysis in comparison with 10/27 (37%) of the vertigo (−) group (*p* = .06).

### 3.4 Relation between the clinical scales and the infarction volume

The total infarction volume did not correlate with NIHSS score on admission (*ρ* = .077, *p* = .56) but correlated with mRS (*ρ* = .37, *p* = .004) as well as Barthel index on discharge (*ρ* = .33, *p* = .011).

## 4 DISCUSSION

### 4.1 Relation of infarction location and volume to vertigo

In the vertigo (+) group, the proportion of men was lower, fewer patients had focal neurological deficits, and the total volume of infarction was larger than in nonvertigo group. Similar to previous studies (Kattah et al., 2009; Kerber et al., 2006; Kim et al., 2017; Neuhauser et al., 2008; Rieger et al., 2014; Saber Tehrani et al., 2014), we found that an infarction location in the cerebellum or dorsal brainstem was significantly related to the development of vertigo. Moreover, the infarction volume, especially for cerebellar infarction, was related to the occurrence of vertigo among our patients. To our knowledge, no similar reports exist in literature. We speculate that larger infarcts, especially in the cerebellum, mediate the development of vascular vertigo through affection of several brain structures and interconnections. On the other side, the development of focal neurological signs results from small strategically located brain infarctions. This might be comparable to patients with small capsular infarctions having moderate or severe hemiparesis, in spite of the small infarction volume. Similar to our results, previous authors identified small lesions ≤10 mm in axial diameter in only 14% of patients with vascular vertigo (Saber Tehrani et al., 2014). The authors found focal neurological signs among 27% of their patients with small lesions, whereby the inferior cerebellar peduncle and the lateral medulla were most often involved. Another study reported vertigo in 92% of patients with lateral medullary infarction plus additional extralateral medullary lesions in comparison with 78.9% of patients with pure lateral medullary infarction (*p* = .008), which is in line with our findings (Kang et al., 2018). Additionally, in one series vertigo was found among all patients with multiple unilateral pontine lesions and in less half of those with a single pontine lesion (Kumral et al., 2002).

### 4.2 Vertigo may mask an underlying vertebrobasilar stroke

In our vertigo (+) group, fewer patients had focal neurological deficits, and hence, the patients tended to present later than the vertigo (−) group. Therefore, numerically fewer patients in the vertigo (+) group received intravenous thrombolysis in comparison with the vertigo (−) group (15.6% vs. 37%, *p* = .06). In concordance with our findings, other authors showed that 37% of patients with vertebrobasilar stroke versus 16% of patients with stroke in anterior circulation were misdiagnosed (*p* < .001) and the presence of focal neurological signs helped to pave the way for the accurate diagnosis (Arch et al., 2016). In a previous cohort, only 42% of patients with vertebrobasilar stroke presenting with vertigo had obvious neurological signs (Kattah et al., 2009). Vertigo was frequently found to be related to misdiagnosis of ischemic stroke (Arch et al., 2016; Newman‐Toker, 2016; Savitz, Caplan, & Edlow, 2007).

### 4.3 Clinical scales and infarction volume

NIHSS score is lower among patients with vertebrobasilar stroke in comparison to those with stroke in the anterior circulation (Inoa, Aron, Staff, Fortunato, & Sansing, 2014; Sarraj et al., 2015; Sato et al., 2008). This can be explained by the fact that several clinical manifestations related to the posterior circulation like vertigo, nystagmus, nausea, or vomiting are not considered in NIHSS (Zuo et al., 2018), but may lead to a worse score on mRS or Barthel index. For example, a patient with vertebrobasilar stroke presenting with severe vertigo may have an NIHSS score of 0 but a mRS score of 3, if he requires some help for the activities of daily living. In our cohort, the infarction volume did not correlate with NIHSS score on admission but correlated with mRS and Barthel index on discharge.

### 4.4 Patient sex, vertigo, and brain infarction

The incidence of stroke, both in the anterior or posterior circulation, in men is around 32% higher than in women (Giroud et al., 2017). Furthermore, the odds ratio for the development of vertebrobasilar stroke rather than stroke in the anterior circulation was even found to be higher in men (Subramanian et al., 2009). Contrarily, men represent around one‐third of the vertigo population (Neuhauser et al., 2008; Rieger et al., 2014). In our cohort, we found less proportion of men among vertigo (+) stroke patients in comparison with vertigo (−) stroke patients. It might be speculated that women were more prone to develop vascular vertigo, and hence, the male proportion among our vertigo (+) stroke patients was diluted. In line with our findings, previous studies found male proportion of 55%–57% among patients with acute vascular vertigo (Kerber et al., 2006; Zuo et al., 2018). In the current work, the infarction volume was insignificantly larger in women. In other terms, the female sex predisposed to vascular vertigo and might have been related to the development of larger infarcts. Similarly, another study showed a negative insignificant association between male sex and infarction volume in the anterior circulation (*p* = .15) (Sun et al., 2016).

### 4.5 One‐fifth of our patients met the old TIA definition of the WHO and at the same time the modern AHA/ASA stroke definition

Historically, the World Health Organization (WHO) defined transient ischemic attack (TIA) as a transient focal neurological deficit lasting <24 hr (Albers et al., 2002). In 2009, the American Heart Association/American Stroke Association (AHA/ASA) revised this definition, stating that TIA is “a transient episode of neurological dysfunction caused by focal brain, spinal cord, or retinal ischemia, without acute infarction” (Easton et al., 2009). The implementation of this AHA/ASA definition “moved” a significant proportion of patients from the TIA to the stroke category (Kvistad et al., 2013). In the current work, 12/59 (20.3%) of the patients had no focal neurological deficits, yet they had a brain infarction on MRI. In other words, those patients met the old TIA definition of the WHO and the modern AHA/ASA stroke definition.

### 4.6 Clinical importance

Our study has several clinical implications. The absence of focal neurological deficits in patients with vertigo should never serve as a differentiating criterion between central and peripheral vertigo. Because of the absence of focal neurological deficits and the delayed presentation, only 15.6% of our stroke patients in the vertigo group received intravenous thrombolysis in comparison with 37% of patients in the nonvertigo group. In the current work, we have shown that the infarction volume was larger in patients with vertigo in comparison to those without vertigo. One of the scenarios commonly encountered in the clinical practice is that of a patient with atrial fibrillation on anticoagulation, who presents with acute onset of vertigo. The clinician is ought to carefully exclude vascular vertigo with possibly underlying large infarction, before continuing the anticoagulation in the acute phase. Otherwise, hemorrhagic transformation may ensue.

Nearly one‐third of nondisabling stroke, especially those with small brain infarctions, those with infarctions in the posterior circulation or with infarctions causing mild perfusion deficits not amounting to induce diffusion‐weighted imaging (DWI) lesion are missed on the initial brain MRI, the so‐called MRI negative stroke (Makin, Doubal, Dennis, & Wardlaw, 2015; Saber Tehrani et al., 2014; Sylaja, Coutts, Krol, Hill, & Demchuk, 2008). We speculate that the absence of vertigo in patients with suspected vertebrobasilar stroke may be a warning sign for the presence of MRI negative stroke.

Whether patients with vertebrobasilar stroke and vertigo or those without vertigo are more likely to benefit from intravenous thrombolysis remains a matter of future research. The presence of large or small infarction may play a role in this regard. Moreover, in patients with wake‐up vertebrobasilar stroke, the infarction volume may affect the clinician's decision to give or refrain from intravenous thrombolysis.

### 4.7 Study limitations

There are limitations of this study. The nonrandomized nature of this single‐center cohort should be kept in mind before a conclusion can be drawn from our results. We recommend the conduction of large randomized multicenter studies in this regard. Another limitation of this study is that the infarction volume and location were assessed by a single nonblinded investigator (AME). However, the assessment methods used in this study are more or less objective.

## 5 CONCLUSION

Cerebellar or dorsal brainstem infarctions are strong predictors of vertigo in vertebrobasilar stroke. Larger infarction volume in these structures may also be related to vascular vertigo. Vertigo without any focal neurological deficit is not uncommon among patients with vertebrobasilar stroke and represents a diagnostic challenge. National institute of health stroke scale has limited value in assessing vertebrobasilar stroke compared with anterior circulation stroke.

## CONFLICT OF INTEREST

None.

## AUTHORS’ CONTRIBUTIONS

All authors made a substantial contribution to the conception, design, and revision of the work. AME and/or FF examined all the patients. AME collected the data, performed the measurements and the statistical analysis, and wrote the first draft. MA, JV, FF, and DE supervised the work, provided consultations, and revised the manuscript. All authors were involved in the final approval of the final version to be published.

## ETHICAL APPROVAL

Data collected within routine clinical care were used. The study was approved from the University Hospital Würzburg Ethics Committee (AZ 223/16). The patients or their next available kin signed an informed consent prior to the inclusion in the study.

## Data Availability

The data that support the findings of this study are available from the corresponding author upon reasonable request.
